# Assessing left atrial function in patients with atrial fibrillation and valvular heart disease using cardiovascular magnetic resonance imaging

**DOI:** 10.1002/clc.23811

**Published:** 2022-03-15

**Authors:** Jie Hou, Yu Sun, Libo Zhang, Wei Wang, Hongrui You, Rongrong Zhang, Benqiang Yang, Huishan Wang

**Affiliations:** ^1^ College of Medical and Biological Informatics Engineering Northeastern University Shenyang China; ^2^ Department of Radiology General Hospital of Northern Theater Command Shenyang China; ^3^ Key Laboratory of Cardiovascular Imaging and Research of Liaoning Province Shenyang China; ^4^ Department of Cardiovascular Surgery General Hospital of Northern Theater Command Shenyang China

**Keywords:** atrial fibrillation, cardiovascular magnetic resonance imaging, feature tracking, left atrial function, strain, valvular heart disease

## Abstract

**Background:**

Atrial fibrillation (AF) is common arrhythmia in valvular heart disease (VHD) and is associated with adverse outcomes.

**Hypothesis:**

To evaluate the left atrial (LA) function in patients with AF‐VHD by cardiovascular magnetic resonance imaging feature tracking (CMR‐FT) using LA strain (*ε*_s_/*ε*_e_/*ε*_a_) and their corresponding strain rate (SRs/SRe/SRa).

**Methods:**

This was a retrospective cross‐sectional inter‐reader and intra‐reader reproducibility conducted from July 1, 2020, to January 31, 2021. A total of 39 patients with AF‐VHD (rheumatic heart valvular disease [RHVD] [*n* = 22], degenerative heart valvular disease [DHVD] [*n* = 17]) underwent MRI scans performed with drug‐controlled heart rate before correcting the rhythm and valves through maze procedure. Fifteen participants with normal cardiac MRI were included as healthy control. *ε*_s_/SRs, *ε*_e_/SRe, and *ε*_a_/SRa, corresponding to LA reservoir, conduit, and booster‐pump function, were assessed using Feature Tracking software (CVI42 v5.12.1).

**Results:**

Compared with healthy controls, LA global strain parameters (*ε*_s_/*ε*_e_/*ε*_a_/SRs/SRe/SRa) were significantly decreased (all *p* < 0.001), while LA size and volume were increased in AF‐VHD group (all *p* < 0.001). In the subgroup, RHVD group showed lower LA total ejection fraction (LATEF) and strain data than DHVD group (12.6% ± 3.3% vs. 19.4 ± 8.6, *p* = 0.001). Decreased LATEF was significantly related to altered LA strain and strain rate, especially in *ε*_s_, *ε*_e_, and SRs (Pearson/Spearman *r/ρ* = 0.856/0.837/0.562, respectively; all *p* < 0.001). Interstudy and intrastudy reproducibility were consistent for LA volumetry and strain parameters (intraclass correlation coefficient: 0.88–0.99).

**Conclusions:**

CMR‐FT can be used to assess the LA strain parameters, and identify LA dysfunction and deformation noninvasively, which could be a helpful functional imaging biomarker in the clinical treatment of AF‐VHD.

## 1 INTRODUCTION

Atrial fibrillation (AF) is the most common human tachyarrhythmia diagnosed clinically; patients with AF are at an increased risk of stroke and heart failure, in addition to a decreased quality of life and lower survival.^1^, ^2^ AF is associated with profound structural and functional alterations of the atrial myocardium,^3^ promoting a true atrial cardiomyopathy, the severity of which is an important determinant of AF recurrence and response to treatment.^4^, ^5^, ^6^ Stroke prevention is a pivotal part of the treatment of patients with AF.^7^ Patients with AF and concurrent valvular heart disease (AF‐VHD) have an even higher thromboembolic risk than those with AF alone.^7^, ^8^ Patients with AF have a 5‐fold increased risk of stroke compared with patients without cardiovascular disease, and patients with AF coupled with mitral stenosis have a 20‐fold risk of stroke.^9^

It is of major clinical interest to have a new imaging biomarker with which to quantify the degree of LA dysfunction and make earlier clinical decisions in patients with AF‐VHD in an effort to prevent cardiac events. The LA normal function includes reservoir (collection of pulmonary venous blood during left ventricular [LV] systole), conduit (passage of pulmonary venous blood flow to the LV during LV early‐diastole), and booster pump function (augmentation of LV filling during LV late‐diastole/atrial systole).^10^ It is important to recognize the interplay that exists between these atrial functions and ventricular performance throughout the cardiac cycle. Previous publications investigating LA function have primarily focused on LA size and volume.^11^ Due to the LA's complex geometry and intricate fiber orientation and the variable contributions of its appendage and pulmonary veins, these two parameters alone may be insufficient to describe the complexity of the LA function and wall motion.^12^, ^13^, ^14^

LA strain assessed by cardiovascular magnetic resonance imaging feature tracking (CMR‐FT) has been used in many cardiovascular diseases and enhanced the diagnostic value,^12^, ^13^, ^14^ which might be higher and more sensitive than conventional LA volumetric parameters^12^ and LV function^13^, ^14^ and presented with good intra‐observer and interobserver reproducibility in normal volunteers.^15^ Due to the very thin LA wall, it is challenging to measure the LA strain.^15^ And radial strain has already been noted for its difficulty to be obtained with poor reproducibility.^15^, ^16^ Echocardiographic data also showed poor reproducibility of strain rate and radial strain^16^ and was limited in the two‐dimensional approaches with a semi‐quantitative and subjective measure.^17^, ^18^ It is clear that studies of LA function provide new insights into the contribution of LA performance to cardiovascular disease and are promising tools for predicting cardiovascular events in a wide range of patient populations. Considerable data also support the use of LAEF for predicting events.^12^ Accordingly, LA function indices such as strain and strain rate have been proposed using noninvasive imaging modalities such as echocardiography speckle tracking^19^, ^20^ and color tissue doppler. Although speckle tracking is presently the only available reference for LA strain estimation, ultrasound beam direction as well as heart motion relative to the probe may influence measurements and inter‐vendor variability, essentially described in the setting of LV function, need to be further investigated.^12^, ^21^

CMR‐FT is a new method to evaluate myocardial strain and strain rate; it can be applied to routine cine images and has the advantages of high spatial resolution, large field of view, good reproducibility, and it can more sensitively reflect the functional characteristics of myocardial tissue.^22^ As a new technique, CMR‐FT has been mainly used in the study of LV strain in recent years,^23^ but has rarely been applied to analysis of LA. The aim of this study was to evaluate LA strain and strain rate using CMR‐FT before valve replacement surgery, assess the feasibility and reproducibility of CMR‐FT for the quantification of global LA function in patients with AF‐VHD, and review the clinical application value of CMR‐FT. In addition, we compared global LA function between patients with AF‐VHD and those without cardiac disease.

## 2 MATERIAL AND METHODS

This study was approved by the local ethics committee. All patients or their families provided written informed consent for the examination.

### 2.1 Patient selection

From July 1, 2020, to January 31, 2021, 39 consecutive patients with AF‐VHD who were admitted to the department of cardiac surgery in our hospital, and performed MRI to assess the left atrium function before valve replacement surgery only or valve replacement surgery with Maze procedure to correct valves and rhythm were included in this study. All patients were with persistent AF and mitral stenosis. The clinical characteristics, echocardiography findings on admission, and cardiac MRI data were retrospectively collected.

The inclusion criteria^24^ were as follows^1^: Patients' age >18 years with AF who were planning to undergo cardiac surgery to correct the rhythm and valves^2^; AF of more than 30 s as recorded by electrocardiogram (ECG) or dynamic ECG; and^3^ patients who were treated according to the American Heart Association/American College of Cardiology/Heart Rhythm Society (AHA/ACC/HRS) AF guidelines for the management of AF^9^ and VHD.^4^, ^25^ According to etiological analysis, the patients divided into two groups, degenerative heart valvular disease (DHVD) group and rheumatic heart valvular disease (RHVD) group. Exclusion criteria included patients who had undergone previous valvular surgery or ablation for AF.

At the same time, 15 healthy participants with normal cardiac MRI in our hospital were included as the healthy control group. The healthy control group excluded claustrophobia, coronary/congenital/valvular heart disease, hypertension, diabetes, severe arrhythmia (e.g., atrial/ventricular arrhythmia, atrioventricular block, pre‐excitation syndrome, and sick sinus syndrome), chronic kidney disease, myocarditis, and cardiomyopathy.

### 2.2 CMR imaging acquisition

CMR studies were performed using a 3.0T MR scanner (Verio, Siemens Medical Systems) and a 32‐channel phased‐array body coil. All images were ECG gated; patients were placed in the supine position and required to hold their breath during image capture. Cine images were acquired in the short‐axis views and longitudinal two‐, three‐ and four‐chamber views using True Fast imaging with Steady‐State Precession (TrueFISP) imaging sequences covering the entire LV and LA (typical field of view 360 × 360 mm, matrix size 216 × 256, slice thickness of 6 mm with no gap, repetition time 40.68 ms, echo time 1.49 ms, flip angle 50°). In addition, we used the cardiac shim model of SIEMENS to adapt adjustment volume to reduce dark band artifacts.

### 2.3 Imaging analysis

Images were analyzed using CVI42 (Circle, version 5.12.1).

**Figure 1:**
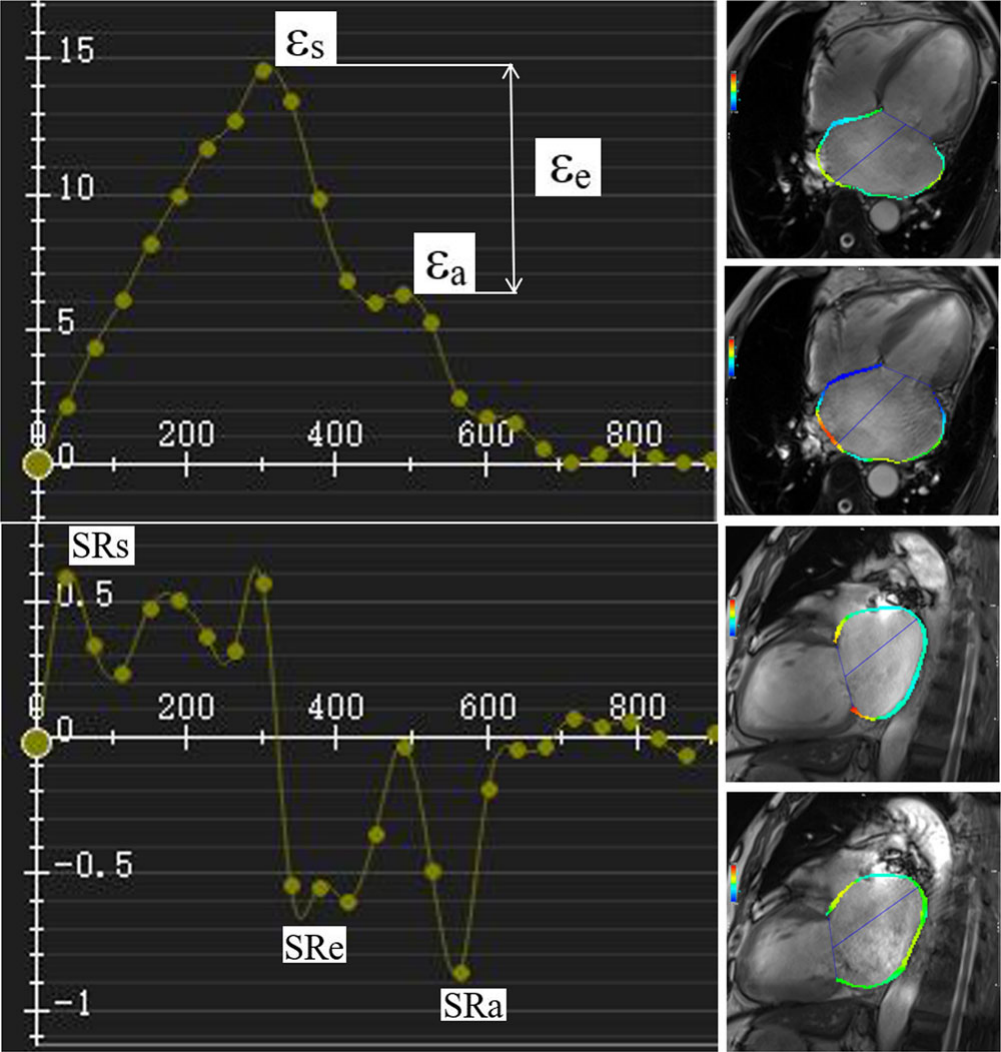
The left atrial (LA) strain and strain rate curve. Global endocardial LA strain and strain rate values (yellow line) were recorded. SRa, peak late negative strain rate; SRe, peak early negative rate; SRs, peak positive strain rate; *ε*_s_, total strain; *ε*_a_, active strain; *ε*_e_, *ε*_s_−*ε*_a_, passive strain. *ε*_s_ and SRs, corresponding to LA reservoir function; *ε*_e_ and SRe, corresponding to LA conduit function; *ε*_a_ and SRa, corresponding to LA booster pump function

(1) LV volumetric parameters: LV end‐diastolic volume (LVEDV, the maximum left ventricular end diastolic filling volume), end‐systolic volume (LVESV, the minimum left ventricular volume at the end of ventricular ejection), ejection fraction (LVEF), cardiac output (LVCO), cardiac index (LVCI) and LV mass (LVM) were measured. LV endocardial and epicardial contours were drawn on LV short‐axis cine images, excluding the papillary muscles. All parameters of LV and LA were corrected according to body surface area (BSA), for example, LVEDV index (LVEDVi) = LVEDV/BSA × 100%, and so forth.
(2) LA strain and strain rate: the LA myocardial deformation was quantified using CVI 42 Tissue Tracking software.^15^, ^26^ LA endocardial and epicardial borders were manually delineated in the apical four‐ and two‐chamber views at end‐diastole using a point‐and‐click approach before the automated tracking algorithm was applied. The pulmonary vein and LA appendage were not included (Figure S1). The software strain analysis model automatically provided the LA strain and strain rate curves. The endocardial LA global longitudinal strain and strain rate values were recorded from the curves^11^: total strain (*ε*_s_), active strain (*ε*_a_), passive strain (*ε*_e_, *ε*_e_ = *ε*_s_−*ε*_a_), peak first positive strain rate (SRs), peak early (first) negative strain rate (SRe), and peak late (second) negative LA strain rate(SRa). *ε*_s_ and SRs, corresponding to LA reservoir function; *ε*_e_ and SRe, corresponding to LA conduit function; *ε*_a_ and SRa, corresponding to LA booster pump function^3^, ^27^(Figure 1).
(3) LA volume (LAV): The LA endocardium was manually labeled by the end‐diastolic atrial minimum volume at the four‐chamber and two‐chamber levels using CVI 42. The pulmonary vein and LA appendage were not included. The parameters of the LA volume were obtained using Simpson's method. The parameters of LAV included the maximum volume (LAV_max_, before the end‐systolic mitral valve was opened) and the minimum volume (LAV_min_, when the end‐diastolic mitral valve was just closed). LA total ejection fraction (LATEF) = (LAV_max_ − LAV_min_)/LAV_max_ × 100%.
(4) The intraobserver and interobserver variability for the LA parameters, measurements were assessed by the intraclass correlation coefficient (ICC).^28^ Intraobserver reproducibility was established by the same observer (J. H., 5‐year experience in CMR diagnosis) who reanalyzed the same subjects after 1 month. Interobserver reproducibility was assessed by a second independent observer (Y. S., 3‐year experience in CMR diagnosis) who was blinded to the first observer's results.

### 2.4 Statistical analysis

Data were analyzed using SPSS, version 20.0 (SPSS Statistics, IBM Corporation). Mean ± SD or median (quartiles) was used to express measurement data in accordance with normal distribution, and the continuous variables were analyzed by independent sample *t*‐test or Mann–Whitney *U* test. *χ*2 or Fisher exact test was used to assess categorical data. Pearson or Spearman correlation was performed to investigate the potential relationship between LA strain parameters and LA function. Moreover, we assessed the ICC to evaluate the accuracy and the precision of the method to measure each LA parameters. ICC was scored as follows: poor reliability, ICC < 0.50; moderate reliability, ICC: 0.50–0.75; good reliability, 0.75–0.9; excellent reliability, ICC > 0.9.^28^ *p*‐value < 0.05 was considered statistically significant.

## 3 RESULTS

### 3.1 Basic demographic characteristics

A total of 39 patients (22 DHVD and 17 RHVD) and 15 control participants were included in the study. Baseline characteristics and volumetric chamber indices for all control participants are summarized in Table 1. The AF‐VHD group had a higher heart rate than the control group (94.8 ± 23.6 bpm vs. 77.3 ± 9.22 bpm; *p *< 0.001), especially in the RHVD group. RHVD group showed lower body mass index (25.8 ± 2.48 kg/m^2 ^vs. 27.4 ± 2.09 kg/m^2^, *p* = 0.048) and BSA (1.84 ± 0.09 m^2 ^vs. 1.92 ± 0.1 m^2^, *p* = 0.015) than DHVD group. RHVD group had more diagnoses of mitral stenosis (21/22 vs. 1/17, *p *< 0.001), and all patients in the AF‐VHD group had mitral regurgitation; there was no statistical significance for tricuspid regurgitation (12/22 *vs*. 12/17, *p* = 0.343). There was no statistical significance for severity of valvular heart disease and proportion of presurgery medications (anti‐platelets, anti‐coagulation, anti‐hypertensive, anticholesterol, hypoglycemics, and antiarrhythmic drugs) between the two groups (all *p >* 0.05).

**Table 1:** Baseline and clinical characteristics of participants

	Control group (*n* = 15)	AF‐VHD (*n* = 39)	*p*	RHVD (*n* = 22)	DHVD (*n* = 17)	*p*
Age (mean ± SD, years)	57.6 ± 9.81	61.9 ± 7.12	0.079	62.3 ± 5.42	61.4 ± 9.02	0.699
Women, *n* (%)	7 (46.7)	23 (59.0)	0.543	16 (72.7)	7 (41.2)	0.059
Body mass index (kg/m^2^)	25.0 ± 3.15	26.5 ± 2.42	0.068	25.8 ± 2.48	27.4 ± 2.09^b^	0.048*
Body surface area (m^2^)	1.79 ± 0.21	1.88 ± 0.09	0.146	1.84 ± 0.09	1.92 ± 0.10^b^	0.015*
Heart rate (bpm)	77.3 ± 9.22	94.8 ± 23.6	<0.001*	100.7 ± 26.5^a^	87.1 ± 17.1	0.061
Cardiovascular risk factors						
Smoking, *n* (%)	‐	11 (28.2)	‐	5 (22.7)	6 (35.3)	0.482
Hypertension, *n* (%)	‐	18 (46.2)	‐	8 (36.4)	10 (58.8)	0.206
Coronary heart disease, *n* (%)	‐	14 (35.9)	‐	5 (22.7)	9 (52.9)	0.091
All types of diabetes mellitus, *n* (%)	‐	6 (15.4)	‐	3 (13.6)	3 (17.6)	>0.99
Prior stroke/TIA history, *n* (%)	‐	10 (25.6)	‐	7 (31.8)	3 (17.6)	0.464
NYHA class on admission ≥Ⅲ, *n* (%)	‐	38 (97.4)	‐	22 (100)	16 (94.1)	0.436
LV function parameters						
LVEDD (mm)	46.6 ± 1.84	50.1 ± 9.78	0.04*	45.6 ± 6.51	55.9 ± 10.4^b^	0.001*
LVEDV (ml)	116.7 ± 21.1	162.7 ± 73.7	0.001*	129.8 ± 44.8	205.3 ± 82.7^b^	0.002*
LVEDVi (ml/m^2^)	66.0 ± 14.2	86.1 ± 36.9	0.006*	70.4 ± 24.9	106.4 ± 40.6^b^	0.001*
LVESV (ml)	46.9 ± 11.2	91.7 ± 41.9	<0.001*	72.4 ± 17.3^a^	116.8 ± 50.9^b^	0.003*
LVESVi (ml/m^2^)	26.5 ± 6.8	48.4 ± 20.5	<0.001*	39.1 ± 9.36^a^	60.4 ± 24.7^b^	0.003*
LVSV (ml)	69.7 ± 13.2	71.1 ± 39.2	0.852	57.5 ± 31.1	88.7 ± 42.3	0.016*
LVSVi (ml/m^2^)	39.5 ± 8.9	37.7 ± 20.5	0.657	31.4 ± 17.3	45.9 ± 21.8	0.031*
LVEF (%)	59.9 ± 5.8	42.6 ± 10.2	<0.001*	42.4 ± 9.75^a^	42.9 ± 11.0^b^	0.877
LVCO (L/min)	5.37 ± 1.13	6.23 ± 2.66	0.102	5.32 ± 1.81	7.42 ± 3.13^b^	0.021*
LVCI (L/min/m^2^)	3.04 ± 0.72	3.31 ± 1.38	0.352	2.89 ± 1.0	3.86 ± 1.63	0.04*
LVM (g)	86.1 ± 19.2	97.5 ± 34.4	0.132	79.4 ± 20.4	120.9 ± 35.0^b^	<0.001*
LVMi (g/m^2^)	47.8 ± 7.43	52.1 ± 16.5	0.189	44.1 ± 10.5	62.6 ± 17.2^b^	<0.001*
VHD						
Mitral stenosis, *n* (%)	‐	22 (56.4)	‐	21 (94.5)	1 (5.9)	<0.001*
Mild/moderate/severe	‐	3/4/15	‐	2/4/15	1/0/0	‐
Mitral regurgitation, *n* (%)	‐	39 (100)	‐	22 (100)	17 (100)	‐
Mild/moderate/severe	‐	10/9/20	‐	10/4/8	0/5/12	‐
Tricuspid stenosis, *n* (%)	‐	0 (0)	‐	0 (0)	0 (0)	‐
Tricuspid regurgitation, *n* (%)	‐	24 (61.5)	‐	12 (54.5)	12 (70.6)	0.343
Mild/moderate/severe	‐	9/10/5	‐	3/5/4	6/5/1	‐
Presurgery medications	‐	‐	‐	‐	‐	‐
Anti‐platelets	‐	10	‐	8	2	0.169
Anti‐coagulation	‐	3	‐	2	1	1.000
Anti‐hypertensive	‐	17	‐	7	10	0.174
Anticholesterol	‐	1	‐	1	0	1.000
Hypoglycemics	‐	3	‐	2	1	1.000
Antiarrhythmic	‐	12	‐	7	5	1.000

*Note*: “*” indicates statistical significance. “a”/“b” indicates statistical significance between the control group and RHVD group/DHVD group.

Abbreviations: AF, atrial fibrillation; CI, cardiac index; CO, cardiac output; DHVD, degenerative heart valvular disease; EDD, end‐diastolic diameter; EDV/ESV(i), end‐diastolic/end‐systolic volume(index); EF, emptying fraction; LV, left ventricular; LVM(i), LV mass(index); NYHA, New York Heart Association; RHVD, rheumatic heart valvular disease; SV(i), stroke volume(index); TIA, transient ischemic attack; VHD, valvular heart disease.

### 3.2 LV parameters

Compared with the control group, the AF‐VHD group showed higher LVEDD, LVEDV(i), LVESV(i), and lower LVEF (59.9% ± 5.8% vs. 42.6 ± 10.2, *p *< 0.001, Table 1). In the RHVD group, LVEDD, LVEDV(i), LVESV(i), LVSV(i), LVCO(LVCI), and LVM(i) were lower than those in the DHVD group. However, there was no significant difference in LVEF between the two groups (42.4% ± 9.75% vs. 42.9 ± 11, *p* = 0.877).

### 3.3 LA structure and function

Patients with AF‐VHD had higher LA size (LAAD and LAV), lower strain values (*ε*_s_/*ε*_e_/*ε*_a_/SRs/SRe/SRa) and LATEF than did control participants (all *p* < 0.001, Table 2). Compared with the DHVD group, the RHVD group showed lower LA strain parameters (*ε*_s_/*ε*_e_/*ε*_a_/SRs/SRe/SRa) and lower LATEF (12.6% ± 3.3% vs. 19.4 ± 8.6, *p* = 0.001).

**Table 2:** Comparison of LA parameters between the control group and patients with AF‐VHD

Mean ± SD/median (IQR)	Control group (*n* = 15)	AF‐VHD (*n* = 39)	*p*	RHVD (*n* = 22)	DHVD (*n* = 17)	*p*
LAAD (mm)	29.9 ± 3.03	53.5 ± 12.4	<0.001*	54.7 ± 12.4^a^	52 ± 12.5^b^	0.502
LAV_max_ (ml)	79 (71–92)	189 (144–244)	<0.001*	210.5 (153–248.5)^a^	182 (137–244)^b^	0.357
LAVi_max_ (ml/m^2^)	45.7 (43–48.6)	102 (79–130)	<0.001*	112.5 (80.75–131)^a^	94 (68.5–127)^b^	0.223
LAV_min_ (ml)	29 (27–33)	162 (126–212)	<0.001*	172 (130.25–214)^a^	137 (110–198.5)^b^	0.174
LAVi_min_ (ml/m^2^)	16.2 (15.4–19.1)	87 (66–110)	<0.001*	98.5 (71–112.25)^a^	73 (56–103)^b^	0.100
LATEF (%)	58.3 ± 2.19	15.6 ± 6.96	<0.001*	12.6 ± 3.3^a^	19.4 ± 8.6^b^	0.001*
*ε*_s_ (%)	38.0 ± 5.39	6.55 ± 6.00	<0.001*	3.97 ± 2.58^a^	9.89 ± 7.48^b^	0.001*
*ε*_e_ (%)	24.4 ± 4.72	5.02 ± 4.21	<0.001*	3.21 ± 2.19^a^	7.36 ± 5.04^b^	0.001*
*ε*_a_ (%)	13.5 (10.8–16.1)	0.7 (0.4–1.9)	<0.001*	0.525 (0.3–1)^a^	1.8 (0.6–3.45)^b^	0.005*
SRs (%/s)	1.9 (1.6–2.2)	0.37 (0.22–0.6)	<0.001*	0.255 (0.2–0.4)^a^	0.6 (0.365–0.85)^b^	<0.001*
SRe (%/s)	−2.2 (−2.6 to −1.8)	−0.28 (−0.43 to −0.13)	<0.001*	−0.2 (−0.31 to −0.1)^a^	−0.42 (−0.665 to −0.23)^b^	0.003*
SRa (%/s)	−2.1 (−2.4 to −1.8)	−0.21 (−0.5 to −0.1)	<0.001*	−0.16 (−0.27 to −0.0875)^a^	−0.45 (−0.85 to −0.215)^b^	0.001*

*Note*: “*” indicates statistical significance. “a”/“b” indicates statistical significance between the control group and RHVD group/DHVD group.

Abbreviations: AF, atrial fibrillation; DHVD, degenerative heart valvular disease; LA, left atrial; LAAD, anteroposterior diameter of left atrium; LATEF, left atrial total ejection fraction; LAV(i)max/min, the maximum/minimum volume of left atrium (index); RHVD, rheumatic heart valvular disease; SRa, peak late negative strain rate; SRe, peak early negative rate; SRs, peak positive strain rate; VHD, valvular heart disease; *ε*_a_, active strain; *ε*_e_, passive strain; *ε*_s_, total strain.

### 3.4 Correlation between strain parameters and LA function in patients with VHD

**Figure 2:**
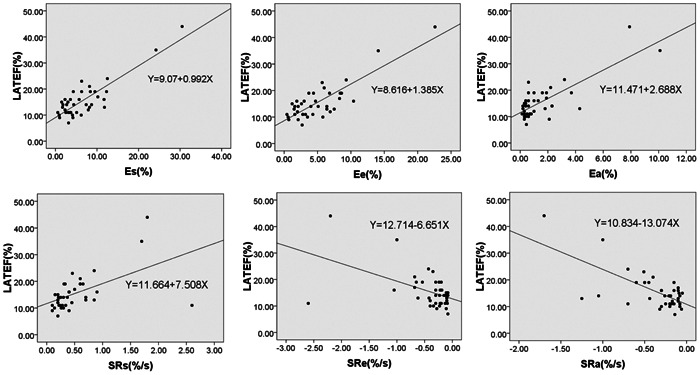
Scatter plots showing correlations of left atrial total emptying fraction (LATEF) and *ε*_s_, *ε*_e_, *ε*_a_, SRs, SRe, and SRa. SRa, peak late negative strain rate; SRe, peak early negative rate; SRs, peak positive strain rate; *ε*_a_, active strain; *ε*_e_, passive strain; *ε*_s_, total strain

LATEF was positively correlated with *ε*_s_, *ε*_e_, *ε*_a_, SRs (*r* = 0.856, *p* < 0.001; *r* = 0.837, *p* < 0.001; *ρ* = 0.501, *p* = 0.001; *ρ* = 0.562, *p *< 0.001; respectively) and negatively correlated with SRe, SRa (*ρ* = –0.407, *p* = 0.01; *ρ* = –0.429, *p* = 0.006; respectively, Figure 2, Table S1).

### 3.5 Reproducibility

Intraobserver and interobserver reproducibility of global LA strain, strain rate, and volumetric parameters using CMR are shown in Table 3. There were excellent and good intra‐observer and interobserver reproducibility, respectively. For intra‐observer reproducibility, the LAV_max_ had the highest reproducibility (ICC, 0.99; 0.98–0.99). For interobserver reproducibility, the LAV_max_ and LAV_min_ had the highest reproducibility (ICC, 0.98; 0.97–0.99). The least reproducible segmental measurement for interobserver reproducibility was the SRe (ICC, 0.88; 0.81–0.93).

**Table 3:** Intraobserver and interobserver repeatability of LA strain and strain rate

	Intraobserver		Interobserver	
	ICC	95% CI	ICC	95% CI
LAV_max_ (ml/m^2^)	0.99	0.98–0.99	0.98	0.97–0.99
LAV_min_ (ml/m^2^)	0.98	0.98–0.99	0.98	0.97–0.99
LATEF (%)	0.96	0.94–0.98	0.95	0.92–0.97
*ε*_s_ (%)	0.98	0.97–0.99	0.97	0.95–0.98
*ε*_e_ (%)	0.96	0.93–0.97	0.96	0.93–0.97
*ε*_a_ (%)	0.97	0.94–0.98	0.95	0.91–0.97
SRs (%/s)	0.90	0.83–0.94	0.89	0.82–0.94
SRe (%/s)	0.89	0.82–0.93	0.88	0.81–0.93
SRa (%/s)	0.97	0.92–0.99	0.94	0.89–0.96

Abbreviations: ICC, intraclass correlation coefficient; LA, left atrial; LATEF, left atrial total ejection fraction; LAV_max_/_min_, the maximum/minimum volume of left atrium; SRa, peak late negative strain rate; SRe, peak early negative rate; SRs, peak positive strain rate; *ε*_a_, active strain; *ε*_e_, passive strain; *ε*_s_, total strain.

## 4 DISCUSSION

Our study evaluated the LA function in patients with AF‐VHD using CMR‐FT. We found that LA function in AF ‐VHD was lower than the Control healthy participants. AF‐VHD patients with reduced reservoir and conduit function, reduced or absent booster pump function, which showed in strain parameters. LATEF has a linear correlation with LA strain parameters. LA enlargement was also observed in AF‐VHD, which means LA remodeling. In the subgroup, DHVD had higher LV size, volume, mass, and stain parameters than those in RHVD. And LA strain and volumetric parameters showed good reproducibility.

LA remodeling consists of both structural and functional changes; enlargement of the LA and fibrosis of the atrial muscle promotes the persistence of AF,^29^ significantly affecting the LA function. The pathophysiology of AF is complex and incompletely understood.^2^ Currently, it is believed that AF‐induced electrical alterations occur within the atrial myocardium (electrical remodeling), which may also promote or accelerate myocardial apoptosis and fibrosis (anatomical remodeling),^2^, ^30^ then the process becomes self‐perpetuating.^31^

LA size by volumetric index is widely accepted as a significant prognostic marker of mortality and outcomes in many cardiovascular diseases.^11^, ^32^ Le Tourneau T et al.^33^ found that patients with organic mitral regurgitation in sinus rhythm who have LAVi ≥ 60 ml/m^2^ and are treated medically have increased mortality and more cardiac events (AF and heart failure). After mitral valve surgery, the cutoff of LAVi ≥ 60 ml/m^2^ lost its prognostic value. Caso P et al.^34^ and Ancona R et al.^35^ showed that the better independent predictor of cardiac events was the SR and *ε*_s_ in patients with asymptomatic rheumatic mitral stenosis followed for 3–4 years. These markers of risk are particularly important because in clinical practice, risk stratification for mitral surgery helps with clinical decision‐making in these patients.^33^ Most of the patients with AF‐VHD in our study had concomitant mitral stenosis and mitral regurgitation and had larger LAV than those of control participants, especially patients with DHVD. Mitral stenosis is associated with LA remodeling, increased LA stiffness, and abnormal atrial contractility. Habibi et al.^36^ found that LA reservoir, conduit function, and booster pump function assessed by CMR‐FT were decreased and negatively correlated with LA myocardial fibrosis in patients with AF. LA strain analysis can detect the existence of a myocardial scar in the early clinical stages,^37^ which can indicate the decrease of LA compliance and indirectly reflect the degree of myocardial fibrosis.^38^ Strain and SR represent the magnitude and rate, respectively, of myocardial deformation.^11^ Interestingly, our results suggest that most of the LA deformation parameters were significantly associated with LATEF, which may suggest the LA strain parameters have a potential correlation with LA wall deformation. Atrial ejection force, the force exerted by LA to accelerate blood into the LV, is another marker of atrial systolic function. LA enlargement and dysfunction (reduced reservoir and conduit function, reduced or absent booster pump function) were found to be present in patients with AF‐VHD, which suggests atrial remodeling and impaired LA myocardial function. It suggested that LA strain with AF‐VHD using CMR‐FT presented impaired strain despite no obvious significant difference in LA volume or function, which may be used in some early‐state cardiovascular patients before obvious changes in volume and function to guide clinical treatment.

Truong VT et al.^15^ using 1.5T MRI and Kowallick JT et al.^39^ using 3.0T MRI found that LA strain and strain rate using CMR‐FT all had good intraobserver and interobserver reproducibility, in addition to good feasibility and reliability. Our study showed the same results. However, Alfuhied A et al.^40^ found that sixty participants including 16 aortic stenosis, 28 type 2 diabetes, 10 end‐stage renal disease on hemodialysis, and 10 healthy volunteers underwent CMR scans, the result showed that LA strain and strain rate assessment using CMR‐FT showed moderate to poor test–retest reproducibility across disease states, LAV and emptying fraction showed more reproducible on CMR. It may be related to the small number and heterogeneous of disease in each group. And all our patients were with mitral valve disease and AF, because the a‐wave peak (*ε*_a_ and SRa) is very low or absent in patients with AF‐VHD,^3^ it is not surprising that the global longitudinal ε_a_ and SRa show good reproducibility.

Previous size (volume) parameters can only reflect the changes in LA structural remodeling, whereas strain parameters can better reflect the early changes in LA in functional parameters. The comprehensive assessment continues to provide more insight into LA function, and therefore more information is available to guide clinical treatment. Future large‐scale studies are warranted to assess whether individual strain parameters using CMR‐FT may provide additional clinical value in stroke risk assessment and guidance of anticoagulation therapy.

## 5 LIMITATION

This was a single‐center study with a relatively modest sample size. We only assessed global longitudinal strain and did not assess radial strain. Radial strain has been noted as a parameter that is difficult to obtain and reproduce consistently.^16^ At present, most research investigates the longitudinal LA strain on the LA long axis, and most of the studies have obtained positive results. It is believed that with the application of three‐dimensional strain analysis technology in the future, its accuracy will be further improved.

## 6 CONCLUSION

CMR‐FT is a reliable tool with good clinical feasibility and repeatability for noninvasive quantitative assessment of LA strain and strain rate (LA function) without using a contrast agent and may provide insight into assessment of the LA performance over time in patients with AF‐VHD, which has potential clinical value in guiding the treatment, prognosis, evaluation and risk stratification.

## CONFLICTS OF INTEREST

The authors declare no conflicts of interest.

## AUTHOR CONTRIBUTIONS

Huishan Wang and Benqiang Yang contributed to the conception and design of the study; Jie Hou, Yu Sun, and Wei Wang contributed significantly to manuscript preparation; Jie Hou and Yu Sun analyzed the data; Jie Hou wrote the manuscript; Libo Zhang, Hongrui You and Rongrong Zhang helped perform the analysis with constructive discussions and revised the manuscript. All authors received the final approval of the submission.

## Supporting information

Supporting information.

## Data Availability

The data underlying this article will be shared on reasonable request to the corresponding author.
