# Supplementary material for: Assessing left atrial function in patients with atrial fibrillation and valvular heart disease using cardiovascular magnetic resonance imaging
Source: Clin Cardiol. 2022 Mar 15;45(5):527–35. doi: 10.1002/clc.23811 (PMC9045075; doi:10.1002/clc.23811)
Supplement: Supplementary file 1 — Supporting information. [file CLC-45-527-s001.doc]

**Supplementary Table 1. Bivariate correlation between LA stain and strain rate and LATEF in patients with AF-VH**D

|  | LATEF | P |
| --- | --- | --- |
| ε_s_ | *r* = 0.856 | P<0.001^*^ |
| ε_e_ | *r* = 0.837 | P<0.001^*^ |
| ε_a_ | *rho* = 0.501 | 0.001^*^ |
| SRs | *rho* = 0.562 | P<0.001^*^ |
| SRe | *rho* = −0.407 | 0.01^*^ |
| SRa | *rho* = −0.429 | 0.006^*^ |

LATEF, left atrial total ejection fraction; ε_a_, active strain; ε_e_, passive strain; ε_s_, total strain; SRa, peak late negative strain rate; SRe, peak early negative rate; SRs, peak positive strain rate. *Indicates statistical significance.


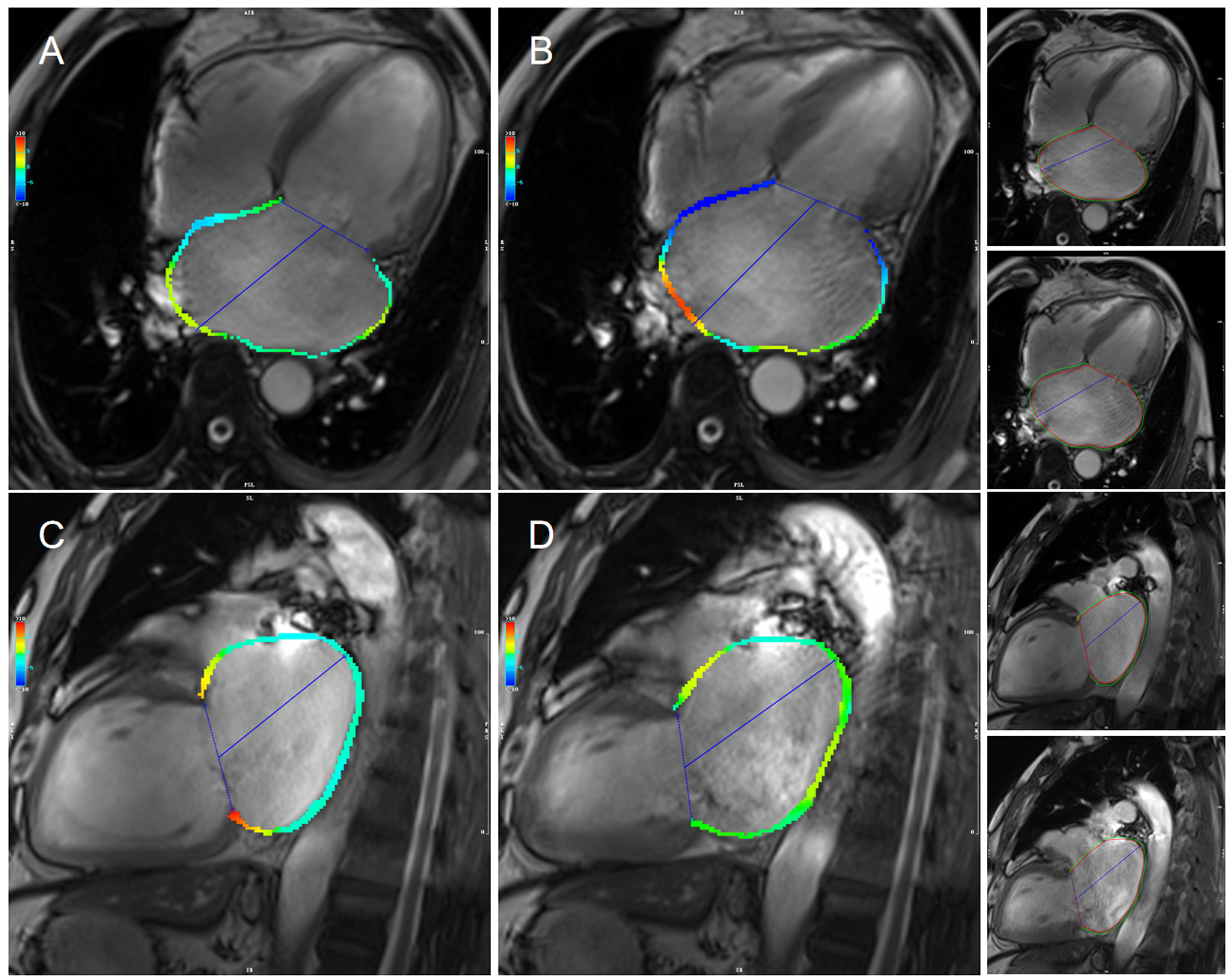


**Supplementary Fig.** LA measurements by CMR feature tracking in patients with degenerative heart valve disease (DHVD). (A and B) LA longitudinal strain in the four-chamber views at end-diastole and end-systole. (C and D) LA longitudinal strain in the two-chamber views at end-diastole and end-systole. On the right side of the figure, the red line represents the endocardial curve, the green line represents the epicardial curve.
